# Impacts of post‐radiotherapy lymphocyte count on progression‐free and overall survival in patients with stage III lung cancer

**DOI:** 10.1111/1759-7714.13621

**Published:** 2020-09-21

**Authors:** Juliana Matiello, Alan Dal Pra, Laise Zardo, Ricardo Silva, Danilo C Berton

**Affiliations:** ^1^ Department of Radiation Oncology Santa Casa de Porto Alegre Porto Alegre Brazil; ^2^ Department of Radiation Oncology UHealth Radiation Oncology Miami Florida USA; ^3^ Department of Pneumological Sciences Universidade Federal do Rio Grande do Sul Porto Alegre Brazil

**Keywords:** Lymphocyte count, non‐small cell lung cancer, progression‐free survival, survival, thoracic irradiation

## Abstract

**Background:**

We evaluated the impact of thoracic radiation in patients with non‐small cell lung cancer (NSCLC), considering the depletion of total lymphocytes, use or not of chemotherapy, and radiation doses in healthy lung tissue.

**Methods:**

Patients with stage III NSCLC, ECOG 0 to 2, receiving radiotherapy with or without chemotherapy were prospectively evaluated. All patients should be treated with three‐dimensional radiotherapy and received biologically effective doses (BED10α/β 10) of 48 to 80 Gy. Peripheral blood lymphocyte total counts were measured at the start of radiotherapy and at 2, 6 and 12 months after radiotherapy. Along with lymphocytes, PTV and doses of 5 Gy and 20 Gy in healthy lung tissue were also evaluated as potential factors influencing overall survival (OS) and progression‐free survival (PFS).

**Results:**

A total of 46 patients were prospectively evaluated from April 2016 to August 2019, with a median follow‐up of 13 months (interquartile range, 1–39 months). The median of OS of all cohort was 22,8 months (IC 95% 17,6–28,1) and the median PFS was 19,5 months (IC 95%: 14,7–24,2). Most patients received concurrent or neoadjuvant chemotherapy (43; 93.4%). No patient received adjuvant immunotherapy. The lower the lymphocyte loss at 6 months after radiotherapy (every 100 lymphocytes/mcL), the greater the chance of PFS (HR, 0.44; 95%CI, 0.25–0.77; *P* = 0.004) and OS (HR, 0.83; 95%CI, 0.70–0.98; *P* = 0.025; *P* = 0.025). BED was a protective factor for both PFS (HR, 0.52; 95%CI 0.33–0.83; *P* = 0.0006) and OS (HR, 0.73; 95%CI 0.54–0.97; *P* = 0.029).

**Conclusions:**

Our results suggest that lymphocyte depletion after radiotherapy reduces tumor control and survival in patients with stage III lung cancer. Radiation doses equal or higher than 60 Gy (BED_10_72 Gy) improve PFS and OS, but they negatively affect lymphocyte counts for months, which reduces survival and the potential of immunotherapy.

**Key points:**

**Significant findings of the study:**

Thoracic irradiation for locally advanced lung cancer depletes T lymphocytes for months. Patients whose lymphocyte loss is lower have better overall survival and progression‐free survival.

**What this study adds:**

It is necessary to protect the lymphocyte population, as well as other organs at risk. New forms of irradiation for large fields are needed. Furthermore, could immunotherapy before chemo‐radiotherapy, with a greater number of lymphocytes, bring an even better result?

## Graphical abstract

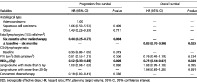

Radiation therapy is an essential local treatment for locally advanced lung cancer; however, the large fields of irradiation may have collateral effects. We revealed that lymphocyte counts decrease for a long time after irradiation, worsening OS and PFS. The other important issue is that low lymphocyte counts may affect the potential of immunotherapy. Immunotherapy at different time points and use of smaller radiation fields with high doses may be key issues for the future of lung cancer treatment.

## Introduction

Most patients with locally advanced non‐small cell lung cancer (NSCLC) have poor outcomes with standard treatment, ie, concurrent or sequential chemoradiotherapy.^1^, ^2^ However, immunotherapy has been used with promising results in the treatment of patients with metastatic and locally advanced disease.^3^

Blockade of programmed death 1 (PD‐1) and programmed death ligand 1 (PD‐L1) has allowed increased local tumor control and survival in patients with lung cancer^4^ but treatment success may be hindered if total and CD4 lymphocyte counts are reduced.^5^ A sufficient lymphocyte count is necessary for PD‐1/PD‐L1 blockade to occur, via immunosuppression, and to allow an increase in immune activity. It has been shown that absolute lymphocyte count <500 cells/mL can compromise the action of drugs that block the PD‐1/PD‐L1 pathway in several tumor types.^6^ Also, a CD4 lymphocyte/total lymphocyte ratio <40% indicates that PD‐1/PD‐L1 blockade therapy has not been successful.^7^ Thus, factors that reduce reduce total lymphocytes count or the percentage of CD4 cells have the potential to reduce or obliterate the response to immunotherapy.

Radiotherapy is known to have the potential to destroy lymphocytes, which are among the most radiosensitive cells in the body. Data from patients irradiated for the treatment of brain, lung and pancreatic tumors have shown that lymphocyte counts remain low long after radiotherapy and negatively impact overall survival (OS) and specific disease in these patients.^8^, ^9^, ^10^ Conversely, there is evidence that radiation might produce neoantigens and stimulate inflammatory factors, increasing the immune response against several tumors.^11^, ^12^ Data from patients undergoing stereotactic radiosurgery have shown a positive impact on the immune response,^13^ and these results indicate that small‐volume, high‐dose radiotherapy may induce synergistic effects on immunotherapy in cancer treatment.

Therefore, the present study aimed to evaluate the effects of the biologically effective dose (BED_10_) on peripheral blood lymphocyte counts (total and fractions) and its impact on OS and progression‐free survival (PFS) in patients with locally advanced, nonmetastatic lung cancer.

## Methods

### Study design

This prospective cohort study was approved by the Research Ethics Committee of Santa Casa de Porto Alegre, Brazil. The study was conducted from April 2016 to August 2019, with follow‐up examinations every two months. Blood samples were collected at the start of radiotherapy for determination of lymphocyte total counts and fractions, and again at two, six, and 12 months after radiotherapy. Changes in total lymphocyte, CD4, CD3, and CD8 counts and in the CD4 lymphocyte/total lymphocyte ratio from baseline (preradiotherapy) to the different post‐radiotherapy time points were evaluated.

### Participants

Written informed consent was obtained from each study participant. Eligible participants were all patients with locally advanced, nonmetastatic NSCLC (stage III according to the American Joint Committee on Cancer seventh edition cancer staging manual).^14^ Patients with an Eastern Cooperative Oncology Group (ECOG) performance status of three or more, previously irradiated patients, and those with a diagnosis of cancer other than NSCLC were excluded.

### Radiotherapy

All participants were treated with three‐dimensional radiotherapy at a dose of 40 to 66 Gy, ranging from 2 to 3 Gy per fraction (BED, 48 to 80 Gy with an α/β ratio of 10), combined or not with chemotherapy. The gross target volume (GTV) was defined as the primary tumor plus involved lymph nodes identified by computed tomography (CT) or positron‐emission tomography (PET). The clinical target volume (CTV) was defined as the GTV plus a margin of 0.5 to 1 cm to cover the microscopic tumor extension. The planning target volume (PTV) included the CTV plus the internal target volume (ITV) of the GTV and an additional margin of 1.0 cm to compensate for setup uncertainties. The radiotherapy plan, including the final dose and the radiation fields, was developed to ensure that the spine cord dose did not exceed 45 Gy, while minimizing the healthy lung dose to the least possible.

All patients were followed clinically every two months until death or the end of the study period. PFS was defined as the time interval between the date of histological diagnosis and the first confirmed sign of local or distant recurrence by imaging and clinical examination. The follow‐up examinations were performed by CT, cranial nuclear magnetic resonance imaging (MRI), and/or PET at the completion of radiotherapy and at two, six, and 12 months after treatment, or earlier if there was evidence of specific symptoms for the assessment of PFS.

### Statistical analysis

Data are expressed as mean and standard deviation (SD) or median and interquartile range (IQR) according to their distribution as assessed by the Shapiro‐Wilk test. PFS and OS survival curves were estimated by the Kaplan‐Meier method. Cox regression models were used to investigate the influence of post‐radiotherapy lymphocyte changes on OS and PFS adjusted for potential confounders. The independent factors previously defined to be evaluated in the model were BED, PTV, age, histological type, and chemotherapy.

Possible associations between lymphocyte changes and PTV, BED, normal lung volume with more than 5 Gy and 20 Gy, and chemotherapy were investigated by Pearson or Spearman's correlation according to the data distribution. The *t*‐test was used to assess differences in lymphocyte counts according to the administration of neoadjuvant or concurrent chemotherapy.

Statistical analysis was performed using SPSS, version 21.0. The level of significance was set at 5%, but variables with *P* < 0.20 in the univariate analysis were included in the multivariate model.

## Results

**Figure 1:**
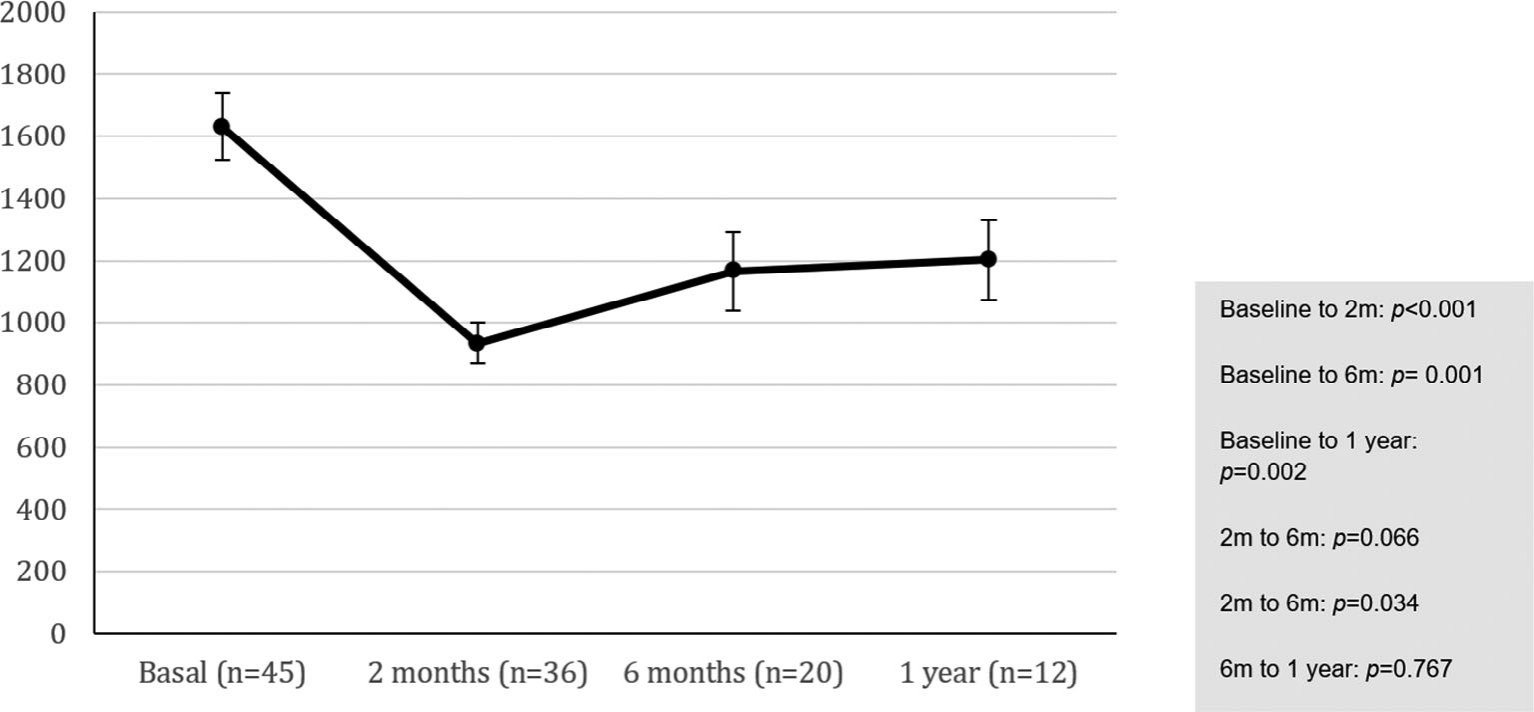
Total lymphocytes versus post‐irradiation time. Data presented as mean ± standard error (mean and 95% confidence interval).

A total of 46 patients were included, with a median follow‐up of 13 months (IQR, 1–39 months). Most participants were in the fifth and sixth decades of life, with a similar number of men and women and a relatively preserved performance status. Most patients received concurrent or neoadjuvant chemotherapy, and the most common regimen was carboplatin and taxane (21; 70%), followed by cisplatin and etoposide (6; 20%), gemcitabine (2; 6.6%), and pemetrexete (1; 3.3%). The median overall survival was 22.8 months (95% confidence interval [CI]: 17.6–28.1) and the PFS 19.7 (95% CI: 14.7–24.2), both measured from diagnosis (Fig 1). Immediately after radiotherapy, most patients had total lymphocyte counts below the reference range (36; 78.2%), but the median counts for lymphocyte subsets were within the reference range (Table 1).

**Table 1:** Baseline characteristics of included patients

Variables	*n* = 46
Age (years) – mean ± SD	62.9 ± 7.1
Sex – *n* (%)	
Male	26 (56.5)
Female	20 (43.5)
Histology – *n* (%)	
Adenocarcinoma	21 (45.7)
Squamous cell carcinoma	18 (39.1)
Other	7 (15.2)
Total lymphocytes (cells/mm^3^) ‐ mean ± SD	1632 ± 741
CD4 (cells/mm^3^) – median (P25–P75)	876.5 (473.5–1062.5)
CD3 (cells/mm^3^) – median (P25–P75)	1188 (779–1585)
CD8 (cells/mm^3^) – median (P25–P75)	363 (258–483.5)
PTV (cm^3^) – median (P25–P75)	532.5 (323.5–930.3)
BED_10_ (Gy) ‐ mean ± SD	72.3 ± 11.8
V5 Gy – mean ± SD	47.7 ± 20.6
V 20 Gy – mean ± SD	22.8 ± 10.6
Concurrent chemotherapy – *n* (%)	30 (65.2)
Neoadjuvant chemotherapy – *n* (%)	13 (28.3)
OS (months) – median (IC 95%)	22.8 (17.6–28.1)
PFS (months) – median (IC 95%)	19,7 (14.7–24.2)
Event – *n* (%)	21 (45.7)
Progression – *n* (%)	15 (32.6)
CNS	6/15 (40)
Local	9/15 (60)
Bony	4/15 (26.7)
Death – *n* (%)	17 (37.0)

BED, biologically effective dose; CNS, central nervous system; P25–P75, 25th–75th percentile; PTV, planning target volume; SD, standard deviation, V5 Gy, lung volume receiving more than 5 Gy; V20 Gy, lung volume receiving more than 20 Gy.

The median baseline total lymphocyte count was 1673 cells/mm^3^ (IQR, 1016–2180 cells/mm^3^). At two months after radiotherapy, total lymphocyte count had a 43% decrease, dropping to a median of 896 cells/mm^3^ (IQR, 682–1186 cells/mm^3^) (*P* < 0.0001), without returning to baseline values at six months (median, 1032 cells/mm^3^; IQR, 696–1.339 cells/mm^3^) and at 12 months (median, 1085 cells/mm^3^; IQR, 897–1.631 cells/mm^3^) (Fig 1). The CD3, CD8, and CD4 lymphocyte subsets also showed a decrease in their counts at two months after radiotherapy (*P* < 0.05), with a nonsignificant increase at six and 12 months.

Therefore, considering six months after radiotherapy as the time point at which lymphocyte counts stabilize (similar to those at 12 months and slightly higher than those at two months), we used this time point to investigate the impact of lymphocyte counts on the primary outcomes (PFS and OS). Regardless of chemotherapy type, BED used, PTV, and histological type, each 100 cells/mm^3^ increase in absolute lymphocyte count at six months after radiotherapy reduced the risk of events by 56% (hazard ratio [HR], 0.44; 95% CI: 0.25–0.77; *P* = 0.0001) at the end of the follow‐up period. Likewise, each 100 cells/mm^3^ increase in the difference in total lymphocyte counts (from baseline to six months) reduced the risk of death by 17% (HR, 0.83; 95% CI: 0.70–0.98; *P* = 0.025) (Table 2).

**Table 2:** Results of multivariate Cox regression analysis for factors associated with progression and death

	Progression‐free survival		Overall survival	
Variables	HR (95% CI)	*P*‐value	HR (95% CI)	*P*‐value
Histological type				
Adenocarcinoma	1.00		—	—
Squamous cell carcinoma	1.66 (0.50–5.53)	0.409	—	—
Other	1.43 (0.22–9.39)	0.711	—	—
Total lymphocytes (100 cells/mm^3^)				
**Six months after radiotherapy**	**0.44 (0.25–0.77)**	**0.004**	—	—
**Δ baseline – six months**	—	—	**0.83 (0.70–0.98)**	**0.025**
CD4/lymphocyte ratio				
Baseline	0.95 (0.86–1.05)	0.319	—	—
PTV (cm^3^) (100 cells/mm^3^)	0.81 (0.55–1.21)	0.306	0.76 (0.49–1.19)	0.234
**BED**_**10**_ **(Gy)**	**0.52 (0.33–0.83)**	**0.006**	**0.73 (0.54–0.97)**	**0.029**
Lung volume with more than 5 Gy	1.00 (0.98–1.03)	0.860	1.08 (0.99–1.18)	0.095
Lung volume with more than 20 Gy	—	—	1.04 (0.85–1.28)	0.691
Concurrent chemotherapy	0.19 (0.00–8.41)	0.386	—	—

BED, biologically effective dose; HR, hazard ratio; PTV, planning target volume; 95% CI, 95% confidence interval.

**Figure 2:**
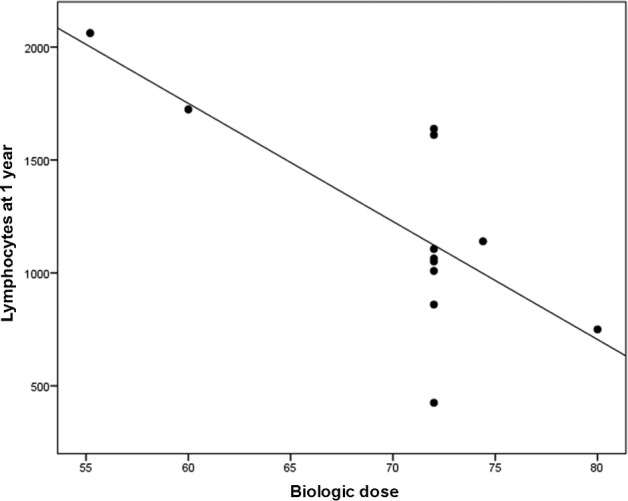
Pearson's correlation between biologic dose and total lymphocytes. r = − 0.732; *P* = 0.007.

BED showed a tendency to be inversely related to lymphocyte count at 12 months (Fig 2). In Fig 2, the results indicate that for large volumes used for the treatment of stage III lung cancer with a median PTV greater than 500 cc, higher BEDs tend to keep the total lymphocyte count below 1000 cells/mm^3^, as analyzed at 12 months after radiotherapy.

## Discussion

The overall survival of all patients was 22.9 months and, although our median was higher than the control arm of the PACIFIC^4^ study 14.6 (95% CI: 10.6–18.6), our data are similar to those of other researchers. Hanna *et al*.^18^ showed a median overall survival of 21.7 months for all patients in their study when they compared the use or not of consolidation docetaxel after chemoradiotherapy for NSCLC stage III. This study showed that the median OS was not better with consolidation docetaxel, with a tendency for patients who did not use consolidation docetaxel to have a longer survival. It is important to mention that the patients in our cohort had only good performance and a high level of education, which were positive prognostic factors for overall survival in the study by Albano *et al*.^19^ Our patients showed a significant decrease in total lymphocyte counts at two, six, and 12 months after radiotherapy with large radiation fields. The mean lymphocyte count at two months was below 1000 cells/mm^3^, exceeding this count only at six months after radiotherapy. Cho *et al*.^5^ showed that patients with metastatic lung cancer treated with immunotherapy had an OS of 5.7 versus 12.1 months (*P* < 0.001) when lymphocyte counts were less than or greater than 1000 cells/mm^3^, respectively.

The median PTV in our sample was 532 cc, including the primary tumor plus involved lymph nodes with CTV and ITV margins, which resulted in a large volume of irradiated blood. Previous studies^15^, ^16^ have shown that it is possible to estimate the amount of irradiated blood through the mean dose volume (in Gy) delivered to the heart, lung, and whole body multiplied by the number of fractions. Jin *et al*.^9^ developed an equation using these data, and the numerical result of this calculation, in Gy, was called the effective dose to the immune cells (EDIC). They reported that an EDIC ≥ 7.3 Gy is associated with a significantly greater reduction in local tumor control, probably due to a decline in lymphocyte counts, which supports the present findings.

In the present study, most patients received concurrent (30; 65%) or neoadjuvant chemotherapy (13; 28.3%). Although only a small number of patients did not receive chemotherapy, our data are consistent with those from larger series showing that neoadjuvant or concurrent chemotherapy was not responsible for long‐term lymphopenia.^17^

The PACIFIC trial, one of the first studies to show relevant data in stage III NSCLC on local tumor control and survival, used immunotherapy after chemoradiotherapy.^4^ Although data on the irradiated volume were not reported, the patients probably received volumes similar to those used in our patients, because like in the present study, only patients with stage III lung cancer were included. Also, usual radiation doses were used, where 99% of patients received a dose of 54 to 74 Gy. Therefore, it can be assumed that there was a significant loss of lymphocytes during chemoradiotherapy, which may have reduced the potential of immunotherapy. The PACIFIC2 trial (ClinicalTrials.gov Identifier: NCT03519971) is currently underway, and the use of immunotherapy combined with chemoradiotherapy at the start of treatment is evaluated in one of the study arms. Early use of immunotherapy may benefit from the presence of more lymphocytes; the results are awaited with interest.

In conclusion, lymphocyte depletion in irradiated patients reduces OS and PFS. Irradiation with higher BEDs (at least BED_10_ of 72Gy) can reduce the number of events and increase survival, but it also reduces lymphocyte count in the long term. With the advent of immunotherapy, it is even more necessary to understand the best time to start radiotherapy and at which volume. The benefits of standard radiation doses or dose escalation without an excessive loss of lymphocytes warrant further investigation in studies with a larger sample size. Immunotherapy at different time points (before, during, or after radiotherapy) and use of smaller radiation fields with high doses may be key issues for the future of lung cancer treatment.

## Disclosure

The authors report no conflict of interest.
